# Integrative Diffusion-Weighted Imaging and Radiogenomic Network Analysis of Glioblastoma multiforme

**DOI:** 10.1038/srep43523

**Published:** 2017-03-07

**Authors:** Dieter Henrik Heiland, Carl Philipp Simon-Gabriel, Theo Demerath, Gerrit Haaker, Dietmar Pfeifer, Elias Kellner, Valerij G. Kiselev, Ori Staszewski, Horst Urbach, Astrid Weyerbrock, Irina Mader

**Affiliations:** 1 Department of Neurosurgery, Medical Center - University of Freiburg, Faculty of Medicine, University of Freiburg, Germany; 2 Department of Neuroradiology, Medical Center - University of Freiburg, Faculty of Medicine, University of Freiburg, Germany; 3 Department of Radiology, University of Basel, Basel, Switzerland; 4 Department of Hematology, Oncology and Stem Cell Transplantation, Medical Center - University of Freiburg, Faculty of Medicine, University of Freiburg, Germany; 5 Medical Physics, Department of Radiology, Medical Center - University of Freiburg, Faculty of Medicine, University of Freiburg, Germany; 6 Department of Neuropathology; Medical Center - University of Freiburg, Faculty of Medicine, University of Freiburg, Germany

## Abstract

In the past, changes of the Apparent Diffusion Coefficient in glioblastoma multiforme have been shown to be related to specific genes and described as being associated with survival. The purpose of this study was to investigate diffusion imaging parameters in combination with genome-wide expression data in order to obtain a comprehensive characterisation of the transcriptomic changes indicated by diffusion imaging parameters. Diffusion-weighted imaging, molecular and clinical data were collected prospectively in 21 patients. Before surgery, MRI diffusion metrics such as axial (AD), radial (RD), mean diffusivity (MD) and fractional anisotropy (FA) were assessed from the contrast enhancing tumour regions. Intraoperatively, tissue was sampled from the same areas using neuronavigation. Transcriptional data of the tissue samples was analysed by Weighted Gene Co-Expression Network Analysis (WGCNA) thus classifying genes into modules based on their network-based affiliations. Subsequent Gene Set Enrichment Analysis (GSEA) identified biological functions or pathways of the expression modules. Network analysis showed a strong association between FA and epithelial-to-mesenchymal-transition (EMT) pathway activation. Also, patients with high FA had a worse clinical outcome. MD correlated with neural function related genes and patients with high MD values had longer overall survival. In conclusion, FA and MD are associated with distinct molecular patterns and opposed clinical outcomes.

Glioblastoma multiforme (GBM) is the most common primary malignant brain tumour in adults, with an incidence of 3–4 cases per 100 000 people^1^. In spite of the best available treatment, the prognosis for patients with GBM is poor, with a median survival of not more than 25–40 weeks^2^,^3^,^4^,^5^,^6^.

“Radiogenomics” is a term used for a research area, which analyses correlations between molecular data from high-throughput techniques (*e*.*g*., whole genome sequencing, gene expression arrays) and the imaging phenotype. Those methods tend to replace the simple analysis of a limited set of genes or pathways. In GBM, this approach was first described by Diehn *et al*. in 2008^7^. This study identified MRI features that significantly correlated to distinct gene expression patterns. Tumours with severe mass effect had an increased expression of genes associated with proliferation. Patients with an infiltrative imaging pattern of perifocal T2-hyperintensity had a worse outcome in comparison to patients with an oedematous appearance. Between both groups, typical mesenchymal or proneural genes (OLIG1, OLIG2, SOX6) were differently expressed^7^. Since then, several studies have investigated the connection between genomics and imaging phenotypes either using a visual pattern evaluation (for a review see Bai *et al*.^8^) or - as recently published - by using texture analysis^9^. However, there is only one radiogenomic study making use of diffusion-weighted imaging in gliomas, which was published by Pope *et al*. in 2012^10^. They correlated transcriptomic data with Apparent Diffusion Coefficient (ADC) values and found 13 genes being significantly correlated to ADC values. Interestingly, 6 of 13 genes were extracellular matrix genes. In addition, patients with low ADC values in the ADC histogram analysis had an improved overall survival^10^.

A few studies analysed correlations between a limited set of genetic alterations and diffusion imaging in gliomas. In 2012, Moon *et al*. used diffusion imaging to discriminate between high grade gliomas with and without MGMT promoter methylation. They found that the ADC values tended to be higher in the methylated than in the unmethylated group. Also, fractional anisotropy (FA) was significantly lower in the methylated group^11^. These results were corroborated by other studies^12^,^13^. In a similar fashion, Young *et al*. found ADC related parameters to be lower in GBM tumours with EGFR amplification as compared to tumours without^14^. In 2014, Cui *et al*. showed that a threshold mean ADC value of 1.565 × 10^−6^ mm^2^/s was able to predict the 1p/19q chromosomal status in WHO II gliomas with 72% sensitivity and 88% specificity^15^. This ADC threshold value was useful for the evaluation of patient outcome as it was able to separate low-grade gliomas into a high- and a low-risk group with acceptable accuracy^15^. Another important molecular characteristic of gliomas is the IDH-status. Wasserman *et al*. identified a significantly higher minimum ADC in IDH1 R132H-mutated tumours as compared to IDH1-wildtype tumours^16^.

Diffusion-weighted MR imaging is often routinely acquired in diagnostic MR investigation of gliomas. Mostly, the ADC is calculated as the average diffusion coefficient from measurements in the x, y and z direction of the magnetic field. When more diffusion directions are measured (≥6 directions), a diffusion tensor can be calculated and further parameters can be deduced. These include:

- axial diffusivity (AD), diffusion coefficient corresponding to the main diffusion direction, parallel to the axonal fibres

- radial diffusivity (RD), diffusion coefficient perpendicular to the main direction

- mean diffusivity (MD), mean coefficient of all diffusion directions

- fractional anisotropy, a measure of the directionality of diffusion

AD is thought to be attributed to axonal integrity and disruption, whereas RD is thought to be associated with changes of the myelin and the environment perpendicular to the nerve fibres^17^. The ADC (being similar to MD) has been discussed to be related to high cellularity, e. g. in gliomas^18^. FA has been described as being reduced in oedema^19^ and necrosis^20^.

To improve our understanding of how gene expression determines the diffusion imaging phenotype, the aim in this work was to provide a comprehensive radiogenomic study using diffusion-weighted imaging parameters in GBM combined with an integrative network analysis of genome-wide expression. This genetic analysis has been chosen instead of focussing only on preselected pathways of interest as it has been done in the past. Furthermore, a connection was sought for between the identified genetic clusters and the classification of GBM by Verhaak *et al*.^21^.

## Results

**Figure 1:**
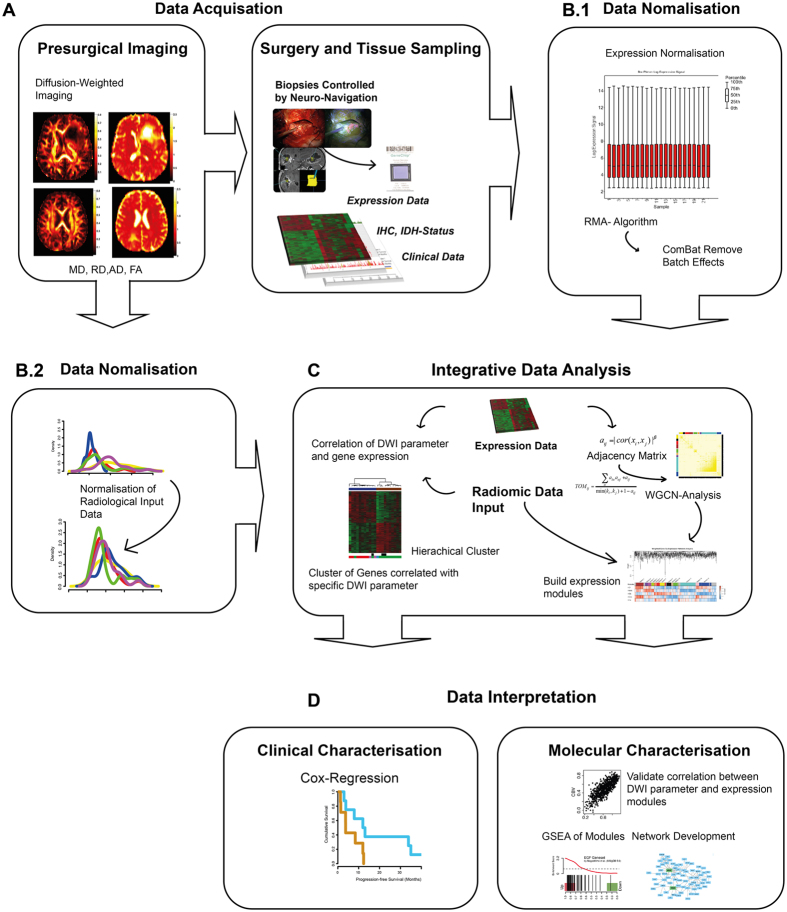
(**A**) The figure shows the workflow and data processing of the “in-house” radiogenomic pipeline. This semi-automated analysis served as a robust method for integrative analysis of imaging and genetic data.

For analysis of the molecular background in tumour dependent diffusion changes, an experimental workflow was developed as presented in Fig. 1. For a better understanding it is shortly summarised hereafter. Four main steps were established in the workflow. First, data were acquired including pre-surgical diffusion weighted imaging (Fig. 1A), followed by a neuronavigated biopsy taken from the contrast-enhancing tumour. In the next step, tissue was prepared and analysed by gene expression array and clinical data were obtained (Fig. 1A). Third, data normalisation of DWI traits (Fig. 1B.2) and gene expression data (Fig. 1B.1) was executed by individual normalisation algorithm as described in the methods part. Then, molecular and imaging data underwent an integrative analysis (Fig. 1C). The main purpose was to characterise each DWI parameter and identify specific underlying molecular functions. The whole bulk of expression data were reduced to expression modules reflecting specific biological functions or pathway activations, using WGCNA (detailed description is given in the methods part). Moreover, these modules were correlated to each DWI parameter to investigate their specific molecular background (Fig. 1D). Additionally, a correlation analysis of all individual genes and DWI parameters was performed by Spearman rank correlation, Fisher’s Exact test and adjusted by Benjamini Hochberg^22^. Genes with a correlation coefficient over/below |−0.6 < 0 < 0.6| were analysed by unsupervised clustering. Given cluster groups reflecting DWI-Radiogenomic subgroups, which were tested by Cox-regression to show differences in their clinical course (Fig. 1D).

### Diffusion Measures

**Figure 2:**
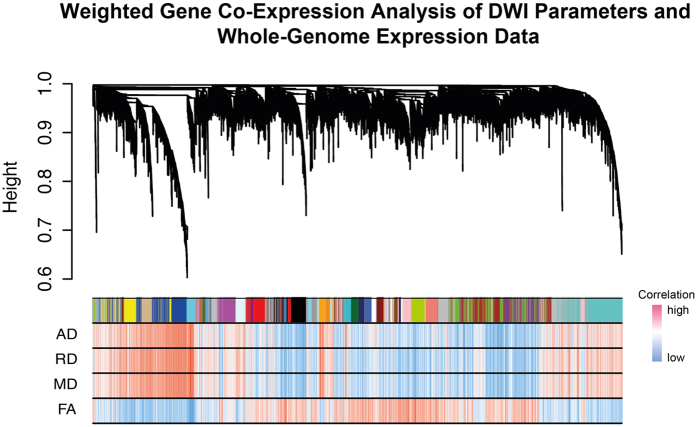
Visualisation of the Weighted Gene Co-Expression Network Analysis. Below the cluster branches, a module bar marks different modules by their specific colours. The correlation heatmap below, indicates the correlation between DWI parameters and respective gene expression. Strong correlation was coloured in red, low correlation in blue.

Figure 2 shows the resulting modules from the WGCNA in the upper part, in association with a heatmap of the correlation between (i) the expression of genes from the entire genome, and (ii) the diffusion measures (AD, RD, MD, FA), in the lower part. One can see that the expression data from the entire genome was sorted into 41 modules by the WGCNA. However, the main observation is that AD, RD and MD correlate with identical genes, while FA correlates with others. We concluded that the parameters AD, RD and MD are subject to a mutual regulation on the gene expression level. Therefore, only MD was considered for further evaluation.

### Mean Diffusivity (MD)

**Figure 3:**
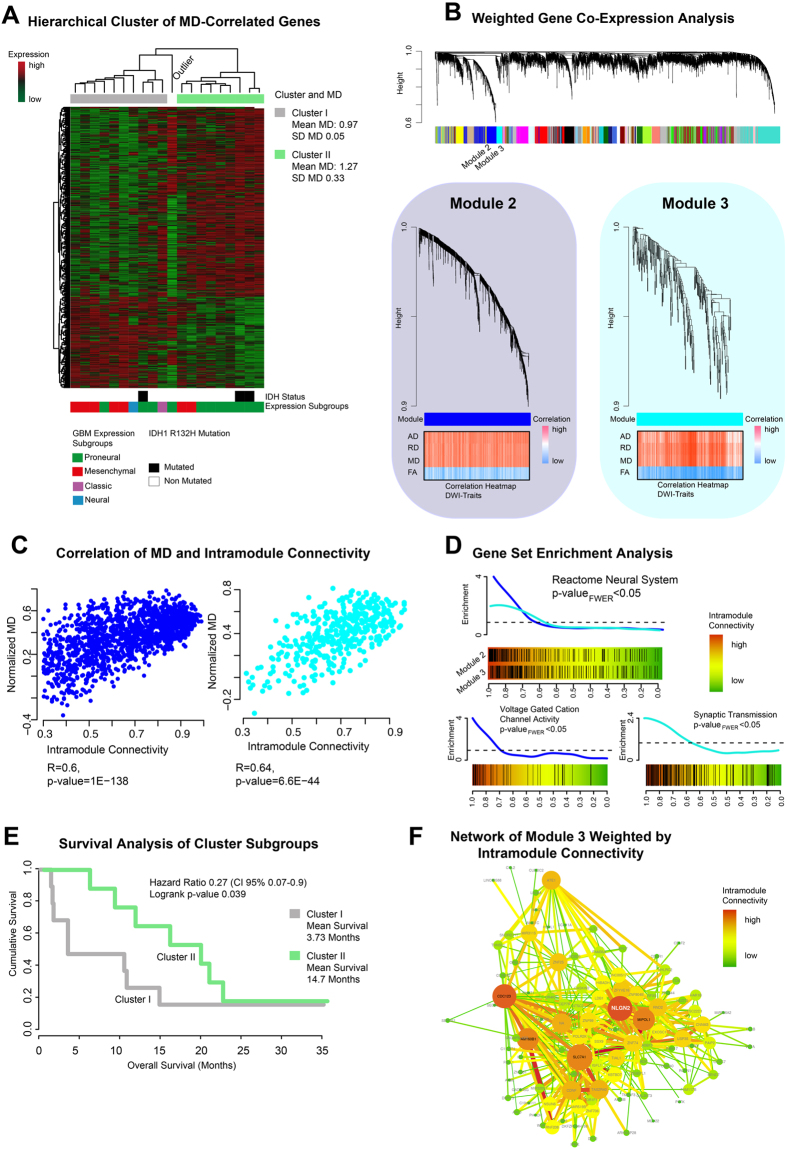
(**A**) Mean diffusivity (MD) associated genes are clustered by Spearman’s rank correlation into two clusters. Bars below the heatmap describe IDH1-status and expression subgroup of each patient. (**B**) Weighted Gene Co-Expression Network Analysis of the whole transcriptomic data. In the bottom panel the detailed branches of module 2 and 3 are presented. Correlation heatmaps of MD and module contained genes are given in the bottom panel. (**C**) Scatterplots of intramodule connectivity (KME) confirmed the strong correlation of MD and module 2 and 3. (**D**) Gene Set Enrichment Analysis of module 2 identified voltage-gated channel activation (bottom left panel), module 3 was associated with synaptic transmission (bottom right panel). (**E**) Survival analysis of patients of cluster I and cluster II (derived from **A**) shows a significantly different OS with a more favourable outcome for the cluster with the higher MD (mean 1.27 ± 0.33) versus the cluster with the lower MD (mean 0.97 ± 0.05), p = 0.039. (**F**) Network analysis of module 3 (derived from (**B**)). Size and colours indicate the intensity of intramodule connectivity.

An unsupervised hierarchical clustering of gene expressions correlating with MD revealed two cluster subgroups: cluster I mean MD: 0.97 ± 0.05 [x 10^−6^ mm^2^/s], cluster II mean MD: 1.27 ± 0.33 [x 10^−6^ mm^2^/s] (Fig. 3A). Both groups were analysed by Kaplan-Meier survival analysis. Patients with high MD in the contrast-enhancing region had a significantly better overall survival (mean OS 14.7 standard deviation 7.49 months) than those with low MD having a worse clinical outcome (mean OS 3.73 standard deviation 6.93 months, p = 0.039) (Fig. 3E).

The WGCNA identified module 2 (ME blue) and 3 (ME cyan) as being highly associated to MD (Fig. 3B). A complete list of correlated modules and diffusion parameters is given in Supplementary Table S2. Further analysis between the intramodule connectivity of module 2 and MD showed a significant correlation of r = 0.6 (p-value < 0.001) (Fig. 3C). In addition, intramodule connectivity of module 3 and MD were strongly correlated r = 0.64 (p-value < 0.001) (Fig. 3C). GSEA was performed to identify biological functions and activated pathways of module 2 and 3 (GSEA in Supplementary Table S3).

Both modules enriched genes attributable to the function of the neuronal system, as listed in the Reactome database (p_FWER_ < 0.001) (Fig. 3D upper row). Tumours with high MD displayed a significantly stronger enrichment of voltage-gated channel activation (p_FWER_ < 0.001, module 2) and of the function of synaptic transmission (p_FWER_ < 0.001, module 3) (Fig. 3D bottom row). A constructed network based on the intramodule connectivity of module 3 is presented in Fig. 3F. Some genes as *NLGN2* are important “hubs” of neural development and neural functions.

### Fractional Anisotropy (FA)

**Figure 4:**
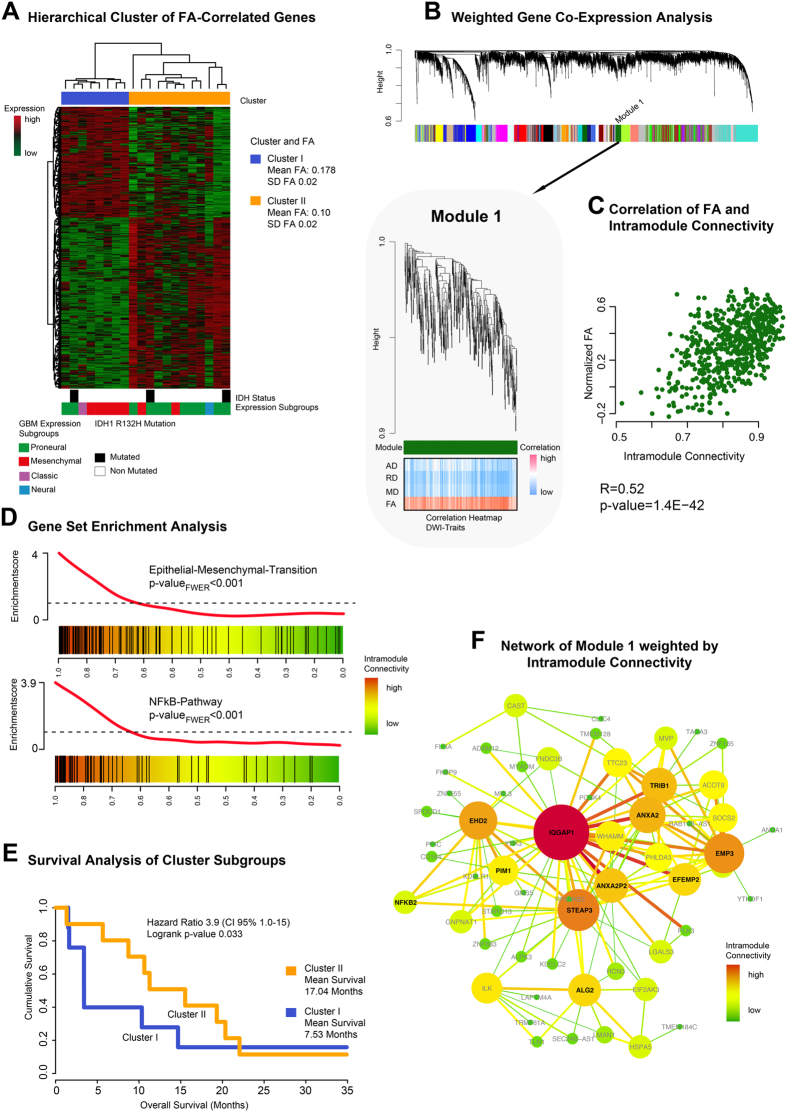
(**A**) Fractional anisotropy (FA) associated genes are clustered by Spearman’s rank correlation into two clusters. Bars below the heatmap describe IDH1-status and expression subgroup of each patient. (**B**) Weighted Gene Co-Expression Network Analysis of the whole transcriptomic data. In the bottom panel the detailed branch of module is presented. The correlation heatmap (bottom panel) shows a strong correlation of module-related genes and FA. (**C**) A Scatterplot of intramodule connectivity (KME) confirmed the strong correlation of FA and module 1. (**D**) Gene Set Enrichment Analysis of module 1 identified epithelial-tesenchymal-Transition (EMT) (upper panel) and NFkB pathway (bottom panel). (**E**) Survival analysis of patients of cluster I and cluster II (derived from (**A**) shows a significantly different OS with a more favourable outcome for the cluster with the lower FA (mean 0.1 ± 0.02) versus the cluster with the higher FA (mean 0.178 ± 0.02), p = 0.033. (**F**) Network analysis of module 1 (derived from (**B**)). Size and colours indicate the intensity of intramodule connectivity.

An unsupervised hierarchical clustering of gene expressions correlating with FA revealed two cluster subgroups: cluster I mean FA: 0.178 ± 0.02 [dimensionless], cluster II mean FA: 0.10 ± 0.02 [dimensionless] (Fig. 4A). Kaplan-Meier survival analysis revealed that patients with low FA in the contrast-enhancing region had a significantly better overall survival (mean OS 17.04 standard deviation 7.70 months) than those with high FA (mean OS 7.53 standard deviation 5.38 months, p = 0.033) (Fig. 4E).

The WGCNA identified module 1 (ME dark green) as being highly associated with the FA (Fig. 4B). Further analysis between the intramodule connectivity and the FA vector depicted a significant correlation of r = 0.52 (p-value < 0.001) (Fig. 4C). GSEA was performed to identify biological functions and activated pathways of module 1 (GSEA in Supplementary Table S3). Tumours with high FA displayed a significantly stronger enrichment of NFκB-pathway activation (p_FWER_ < 0.001) and higher expression of genes attributable to the epithelial-mesenchymal-transition (EMT) than those with a low FA (p_FWER_ < 0.001, Fig. 4D). A constructed network based on the intramodule connectivity of module 1 is presented in Fig. 4E, and shows some hub-genes as *IQGAP1, ANXA2* and *EMP3* being important “leaders” of the related pathway activation.

## Discussion

This study aimed at performing a genome-wide profiling of expression data and pathways related to diffusion-weighted imaging. Network-based data integration of transcriptomic data and imaging traits was used to highlight the biological background of the diffusion parameters MD and FA.

### Mean Diffusivity

Kallenberg *et al*. showed that a small increase of ADC in the contralateral normal appearing matter is predictive of microchanges of normal tissue suggesting tumorous transformation^23^. So far, ADC as simplified form of MD is regarded as a sensitive parameter for microstructural changes due to tumour infiltration.

In our study, MD was associated with two modules of the WGCNA. Both modules were related to neural functions (Fig. 3). Module 3 was highly associated with synaptic transmission as shown in Fig. 3D. In addition, different hub-genes such as *NLGN2* were identified. Neuroligin (*NLGN*) belongs to a group of proteins associated with neural development and genetic alterations found primarily in autism^24^. *NLGN3* is associated with increased tumour growth of glioblastoma^25^. Stimulation of NLGN leads to an activation of neural precursor cells (NPCs), and NLGN proteins support their growth. In gliomas, this mechanism activates the oncogenic AKT pathway and supports tumour growth and invasiveness^25^.

An increased MD is thought to be attributed to interstitial oedema. So tumours with more oedema had a better survival than those without oedema in this study. This finding is corroborated by the study of Diehn *et al*. who identified an oedematous (versus infiltrative) pattern of T2-hyperintensity on structural MR imaging that was also related to a better prognosis (LIT).

### Fractional Anisotropy

Fractional anisotropy (FA) mirrors the degree of anisotropy in a given tissue. This study focused on FA changes in the contrast enhancing region of different GBMs and their associated transcription profiles. FA was associated with module 1 of the WGCNA. This module contained genes associated with the epithelial-mesenchymal transition pathway. This oncogenic pathway is well known in different types of cancer and is one of the hallmarks of oncogenic transformation^26^,^27^,^28^,^29^,^30^. Moreover, NFκB-pathway activation was enriched in module 1. The activation of the NFκB-pathway and up-regulation of NFκB related genes supports EMT in gliomas and is associated to the mesenchymal expression subtype^31^,^32^. A further network analysis of module 1 presented different hub-genes such as *IQGAP1, ANXA2* and *EMP3*. These genes are additionally contained in the mesenchymal signature described by Verhaak^21^ and are assumed to have an important function in the EMT^26^,^33^,^34^,^35^,^36^. Most of the samples containing a low FA were identified as proneural samples and showed a significantly better clinical outcome. However, the findings of Jiang *et al*., who showed that FA was negatively correlated with KI-67^37^, a marker of mitosis and hence aggressiveness, seem contradictory. In summary, FA does not seem to be merely attributable to quantitative measures of cellular architecture, but also seems to be related to the expression of specific biological pathways such as the EMT.

### Limitations

The main limitation of this study is the small number of cases. However, conservative statistical methods with corrections for multiple testing at each level of analysis were applied. Only family-wise error corrected values are reported for the sake of robustness in GSEA.

In conclusion, the presented work is a radiogenomic study of glioblastoma multiforme involving an integrative analysis of diffusion-weighted imaging and genome-wide expression profiling. High FA was associated with a worse clinical outcome and activation of the EMT whereas high MD was correlated with better overall survival and increased expression of neural function genes.

## Material and Methods

### Patients

For this prospective study we included 21 patients (median age 66 years, range 41–84 years) who underwent surgery in the department of neurosurgery between 2012 and 2014. The local ethics committee of the University of Freiburg approved data evaluation, imaging procedures and experimental design (protocol 100020/09 and 5565/15). The methods were carried out in accordance with the approved guidelines. Written informed consent was obtained from all patients.

Inclusion criteria were: (1) age older than 18 years, (2) preoperative MRI with diffusion-weighted imaging, (3) intraoperative MRI-guided sampling of tumour tissue from contrast-enhancing tumour, (4) histopathological confirmation of a glioblastoma multiforme (WHO criteria). 21 patients could be enrolled into this study (Supplementary Table S1).

### Tissue collection and histology

Tumour tissue was sampled from contrast enhancing regions identified by intraoperative neuronavigation (Cranial Map Neuronavigation Cart 2, Stryker, Freiburg, Germany) during tumour resection. The tissue was snap-frozen in liquid nitrogen immediately and processed for further genetic analysis. Tissue samples were fixed using 4% phosphate buffered formaldehyde and paraffin-embedded with standard procedures. H&E staining was performed on 4 μm paraffin sections using standard protocols. Immunohistochemistry was applied using an autostainer (Dako) after heat-induced epitope retrieval in citrate buffer. IDH1 mutation was assessed by immunohistochemistry using an anti-IDH1-R123H antibody (1:20, Dianova).

### MR-Imaging

MR imaging was performed on a 3T system (Magnetom TIM TRIO, Siemens, Erlangen, Germany) using a 12-channel head coil. The imaging protocol consisted of a 3D T2-weighted fluid attenuated sequence (repetition time (TR), 5,000 ms; effective echo time (TE_eff_), 388 ms; inversion time (TI), 1,800 ms; flip angle, variable; pixel size; 1 mm^3^), a 3D T1-weighted magnetisation prepared rapid gradient echo sequence (TR, 1390 ms; TE, 2.15 ms; TI, 800 ms; flip angle, 15°; pixel size; 1 mm^3^) was acquired with application of 0.1 mmol/kg body weight Gadobenate Dimeglumin (Multihance^®^, Bracco, Konstanz, Germany). For diffusion imaging, a diffusion sensitive single-shot spin-echo EPI sequence with distortion correction^38^ was applied (61 diffusion encoding gradient directions; b-value, 0, 1000 s/mm^2^; TR, 8,800 ms; TE, 102 ms; pixel size, 2 × 2 × 2 mm^3^).

### MRI Post-Processing

All imaging data were co-registered in individual space by using spm8 (http://www.fil.ion.ucl.ac.uk/spm/software/spm8/) to reduce effects of motion. Diffusion data were processed by using a MATLAB-based in-house toolbox for fibre tracking (https://www.uniklinik-freiburg.de/mr-en/research-groups/diffperf.html). The effective self-diffusion tensor was computed on diffusion data corrected for motion and distortion artefacts. Maps of axial (AD), radial (RD) and mean diffusivity (MD) and fractional anisotropy were calculated in contrast enhancing tumour. A manual region-of-interest evaluation was performed for the whole contrast enhancing tumour, carefully excluding cerebrospinal fluid and arterial and venous vessels. The median value was chosen for further evaluation.

### Genome-Wide Expression Analysis

RNA was prepared using the RNAeasy kit (Qiagen). An amount of 1.5 μg RNA was obtained for expression array analysis. Arrays were performed using human genome 2.0 chips (Affymetrix). Raw data were processed, normalised and controlled by R software and the Affymetrix R-package. Different expression analysis and statistical testing (pairwise t-test) were performed by limma R-package. Additional information was given in the supplementary file (Supplementary - Methods).

### Weighted Gene Co-Expression Network Analysis

WGCNA uses the topological overlapping measurement to identify corresponding expression modules. These expression modules were analysed by their eigengene correlation to each diffusion parameter. The WGCNA analysis is a robust tool for integrative network analysis and was used in several recent studies^39^,^40^,^41^. In addition, a permutation-based pre-ranked Gene Set Enrichment Analysis (GSEA) was applied to each expression module to verify its pathways^42^. The predefined gene sets of the Molecular Signature Database v5.1 were taken. Networks were exported to Cytoscape 2.0^43^ for further visualisation. The WGCNA integrated function (exportNetworkToCytoscape) was used to calculate a weighted network. A detailed description of WGCNA was given in Heiland *et al*.^44^. Additional information was given in the supplementary file (Supplementary - Methods).

### Gene Set Enrichment Analysis (GSEA)

Permutation based gene set enrichment analysis (GSEA) was performed for each expression module to find specifically enriched biological functions and related pathways^42^. Pre-ranked GSEA was performed with 1000 permutations. P-values were calculated by familywise error rate (FWER) which is a robust method for multiples testing^22^. The Molecular Signatures Database version 5.0 was used including pathways gene sets (C2) (http://www.broadinstitute.org/gsea) as input databases for this analysis. GSEA plots were visualised by limma R-package (barcodeplot function). Additional information was given in the supplementary file (Supplementary - Methods).

### Survival Analysis

Progression-free survival (PFS) was available for 18/21 patients. The other three patients had no tumour progression until the end of 2015. For 16 patients overall survival was available, whereas 5 patients are still alive and have outstanding overall survival (OS). The Kaplan-Meier method was used to provide median point estimates and time-specific rates. The Hazard-Ratio (HR) was calculated using Cox-Regression tests.

## Additional Information

**How to cite this article**: Heiland, D. H. *et al*. Integrative Diffusion-Weighted Imaging and Radiogenomic Network Analysis of Glioblastoma multiforme. *Sci. Rep.* **7**, 43523; doi: 10.1038/srep43523 (2017).

**Publisher's note:** Springer Nature remains neutral with regard to jurisdictional claims in published maps and institutional affiliations.

## Supplementary Material

Supplementary Data
