# Supplementary material for: Integrative Diffusion-Weighted Imaging and Radiogenomic Network Analysis of Glioblastoma multiforme
Source: Sci Rep. 2017 Mar 7;7:43523. doi: 10.1038/srep43523 (PMC5339871; doi:10.1038/srep43523)
Supplement: Supplementary Data [file srep43523-s1.pdf]

Integrative Diffusion-Weighted Imaging and Radiogenomic Network Analysis of Glioblastoma multiforme (Supplementary)

Dieter Henrik Heiland[1], Carl Philipp Simon-Gabriel[2], Theo Demerath[2,6], Gerrit Haaker[1], Dietmar Pfeifer[3], Elias Kellner[4], Valerij G. Kiselev[4], Ori Staszewski[5], Horst Urbach[2], Astrid Weyerbrock[1], Irina Mader[2]

[1]Department of Neurosurgery, Medical Center - University of Freiburg, Faculty of Medicine, University of Freiburg, Germany

[2]Department of Neuroradiology, Medical Center - University of Freiburg, Faculty of Medicine, University of Freiburg, Germany

[3]Department of Hematology, Oncology and Stem Cell Transplantation, Medical Center - University of Freiburg, Faculty of Medicine, University of Freiburg, Germany

[4]Medical Physics, Department of Radiology, Medical Center - University of Freiburg, Faculty of Medicine, University of Freiburg, Germany

[5]Department of Neuropathology; Medical Center - University of Freiburg, Faculty of Medicine, University of Freiburg, Germany

[6]Department of Radiology, University of Basel, Basel, Switzerland

Corresponding author:
Dieter Henrik Heiland
Department of Neurosurgery
Medical Center - University of Freiburg
Faculty of Medicine, University of Freiburg
Breisacher Strasse 64
D-79106 Freiburg
-Germany-
Tel: +49 (0) 761 270 50010
Fax: +49 (0) 761 270 51020
E-mail: dieter.henrik.heiland@uniklinik-freiburg.de

Supplementary

Methods

Preparing Expression Data

RNA was prepared using the RNAeasy kit (Qiagen). An amount of 1.5 µg RNA was obtained for expression arrays analysis. Arrays were performed by human genome 2.0 chip (Affymetrix). Raw data were processed, normalised (Robust Multi-array Average (RMA)) and controlled by R software and the Affymetrix R-package.

Correlation Analysis of Gene Expression and DWI Parameter

Correlation of each DWI parameter and expression data was performed by pairwise spearman correlation of each gene and DWI parameter. Significance was tested by Fischer's Exact test and adjusted for multiples testing by Bejamini Hochberger [3]. In a next step, significant correlated genes ($p<0.05$) were analysed by an unsupervised hierarchical clustering (Figure 3A, 4A). For a detailed analysis of molecular functions and pathway activation, we added a network-based analysis.

Weighted Gene Co-Expression Network Analysis (WGCNA)

The WGCNA is a tool first described by Steve Horvath and Peter Langfelder[18]. It is a powerful and robust tool to analyse preselected gene sets and find correlated biological functions or related pathways to specific clinical traits. In our case, we integrated our DWI parameter as input for this analysis. The WGCNA tool is available on the bioconductor database and processed in R-software. All expression data were used as input for WGCNA analysis. In a first step, outliers in the expression dataset were identified by a normalised connectivity below the threshold of -2.5. All samples identified as outliers were excluded from further analysis; no outlier was identified in our dataset.

The following R-code was used:

```
A=adjacency(t(datExpr),type="distance")

k=as.numeric(apply(A,2,sum))-1
```

```
Z.k=scale(k)
thresholdZ.k=-2.5
```

In a next step, further analysis was performed as signed network analysis with a soft threshold (power ß) based on scale-free topology criteria. We tested different ß-values by following r-function.

The following R-code was used:

```
powers=c(1:20)
sft=pickSoftThreshold(datExpr,powerVector=powers, networkType = "unsigned")
```

Calculation of SFT:

$$R_{SFT} = |cor(\log(freq_{KN}), \log(KN))| > 0.9$$

If the correlation between the logarithm of connectivity (KN) and frequency of connectivity ($freq_{KN}$) achieved values above 0.9, we assume that SFT criteria are adhered. The calculated power was implemented in the WGCNA analysis. In addition, dynamic tree cutting was used as described by Peter Langfelder[14] with a deep split of two.

Characterising of Expression Modules

Given modules as presented in Figure 3B and 4B with a significant positive correlation to each module eigengene were used for further validation. Additionally, another parameter was used for validation: The “intramodule connectivity (kME)”. kME ranked all genes in a module by its “importance” (connectivity) and was correlated to DWI parameters (Figure 3C, 4C).

The following R-code was used:

```
datKME=signedKME(datExpr, ME)
```

In a next step, gene set enrichment analysis investigated pathway functions and biological background of each expression module. Permutation based gene set enrichment analysis (GSEA) was performed

for correlated module to find specifically enriched biological functions and related pathways [2]. Pre-ranked GSEA were performed with 1000 permutations. P-values were calculated by familywise error rate (FWER) which is a robust method for multiples testing [3]. The Molecular Signatures Database version 5.0 was used including pathways gene sets (C2) (http://www.broadinstitute.org/gsea) as input databases for this analysis. GSEA plots were visualised by limma R-package (barcodeplot function). All genesets (C2 all) were tested without pre-analytic sorting out specific genesets (Figure 3D and 4E).

References:

1. Langfelder, P. & Horvath, S. WGCNA: an R package for weighted correlation network analysis. *BMC Bioinformatics* **9,** 559 (2008).
2. Subramanian, A. *et al.* Gene set enrichment analysis: a knowledge-based approach for interpreting genome-wide expression profiles. *Proc. Natl. Acad. Sci. U. S. A.* **102,** 15545–50 (2005).
3. Benjamini, Y. & Hochberg, Y. Controlling the false discovery rate: a practical and powerful approach to multiple testing. *Journal of the Royal Statistical Society* **57,** 289–300 (1995).

Tables:

Supplementary Table S1

Patient	Overall Survival	PFS	IDH1-Mutation	Radial Diffusivity [x 10-6 mm2/s]	Axial Diffusivity [x 10-6 mm2/s]	Mean Diffusivity [x 10-6 mm2/s]	Fractional Anisotropy [dimensionless]	Expression Subgroup
Pat_GBM_1	alive	33.84	wildtype	0.81	1.09	0.9	0.22	Mesenchymal
Pat_GBM_2	1.64	1.25	wildtype	0.77	1.11	0.87	0.21	Mesenchymal
Pat_GBM_3	alive	no PFS	wildtype	0.91	1.24	1.01	0.19	Proneural
Pat_GBM_4	11.34	2.97	wildtype	0.98	1.27	1.07	0.18	Mesenchymal
Pat_GBM_5	16.1	8.36	wildtype	0.87	1.15	0.96	0.18	Mesenchymal
Pat_GBM_6	3.72	3.72	R132H Mutation	0.94	1.2	1.03	0.17	Proneural
Pat_GBM_7	3.72	3.72	wildtype	0.94	1.2	1.03	0.17	Mesenchymal
Pat_GBM_8	11.67	9.15	wildtype	0.88	1	0.94	0.16	Neural
Pat_GBM_9	6.2	3.84	wildtype	0.93	1.19	1.01	0.15	Mesenchymal
Pat_GBM_10	12.36	12.36	wildtype	1.06	1.42	1.18	0.15	Proneural
Pat_GBM_11	1.77	1.77	wildtype	1.09	1.37	1.19	0.15	Classic
Pat_GBM_12	1.44	1.41	wildtype	0.92	1.12	0.99	0.15	Proneural
Pat_GBM_13	22.34	13.03	wildtype	1.05	1.28	1.12	0.14	Proneural
Pat_GBM_14	alive	no PFS	wildtype	1.13	1.36	1.21	0.13	Classic
Pat_GBM_15	alive	no PFS	R132H Mutation	1.21	1.45	1.29	0.12	Proneural
Pat_GBM_16	24.2	12	wildtype	1.07	1.29	1.14	0.11	Proneural
Pat_GBM_17	21.21	7.43	wildtype	1.07	1.28	1.14	0.11	Mesenchymal
Pat_GBM_18	9.57	7.87	wildtype	1.15	1.35	1.22	0.1	Proneural
Pat_GBM_19	3.73	3.73	wildtype	1.04	1.21	1.09	0.09	Proneural
Pat_GBM_20	alive	15.3	R132H Mutation	1.32	1.5	1.37	0.08	Proneural
Pat_GBM_21	17.04	10.82	wildtype	2.25	2.42	2.3	0.04	Proneural

Supplementary Table S2

modTrait	AD	RD	MD	FA
MEsteelblue	-0.531541206	-0.645146415	-0.604735109	0.727126769
MEmidnightblue	-0.357348538	-0.51161527	-0.470806766	0.576809068
MEgreenyellow	-0.349503233	-0.469404123	-0.423309201	0.549853878
MEsalmon	-0.229927999	-0.385365215	-0.326761407	0.528940712
MEplum1	-0.279401992	-0.336852385	-0.302050905	0.525202026
MEpurple	-0.544690469	-0.614902831	-0.609742273	0.499355525
MEpink	-0.226581017	-0.383781685	-0.330771084	0.483882738
MEdarkgreen	-0.303326611	-0.368192959	-0.35021039	0.47746196
MEroyalblue	-0.063497438	-0.2573752	-0.205058281	0.457145796
MElightyellow	-0.151966298	-0.30078578	-0.254773662	0.438947732
MEsaddlebrown	-0.217589344	-0.28574029	-0.247170399	0.405013402
MEdarkred	-0.232782189	-0.311886369	-0.29252715	0.391175444
MEgreen	-0.587795817	-0.537589231	-0.548703971	0.384622197
MElightgreen	-0.414748532	-0.428370816	-0.438079988	0.357398364
MEgrey	-0.14807133	-0.273094241	-0.252928302	0.342221151
MEblack	-0.510269116	-0.467409232	-0.485074935	0.33369956
MEgrey60	-0.251605764	-0.317031375	-0.286417922	0.327763714
MEred	-0.261158696	-0.306089356	-0.294593158	0.318477905
MEwhite	0.130506479	-0.044451656	0.000568281	0.303064511
MEyellowgreen	0.141229496	-0.106725639	-0.045692698	0.238412545
MEdarkturquoise	0.107945948	-0.050498487	-0.012470312	0.23013308
MEdarkorange	0.279163842	0.113123647	0.164715574	0.159045434
MEmagenta	0.007186125	-0.021836781	-0.003966607	0.081571107
MEdarkmagenta	0.100663044	0.045643136	0.043197178	0.04400239
MEbrown	-0.394002218	-0.264667338	-0.307470189	0.004193201
MEpaleturquoise	0.231093962	0.23609991	0.257419196	-0.027690469
MEskyblue	-0.19044115	-0.091687954	-0.113321703	-0.080759733
MEviolet	-0.125527315	-0.016128546	-0.06296557	-0.169650636
MEdarkolivegreen	-0.031867578	0.101750095	0.050842138	-0.213031789
MEskyblue3	-0.009904185	0.091608751	0.060659604	-0.227067426
MEsienna3	0.323118174	0.329730304	0.326901809	-0.338400971
MEorangered4	0.287260246	0.368184276	0.352684921	-0.38214814
MEturquoise	0.145093013	0.242054441	0.220905933	-0.388150283

MElightcyan	0.297284352	0.384880132	0.368161252	-0.394188304
MEorange	0.590853223	0.61353054	0.620728934	-0.398525104
MEblue	0.540380875	0.55778475	0.555976295	-0.417257671
MEdarkgrey	0.476831115	0.531144731	0.527997006	-0.442687688
MEtan	0.409621375	0.519702111	0.484963768	-0.52455476
MEyellow	0.329679459	0.46877193	0.418572637	-0.58002453
MEcyan	0.498212658	0.636474975	0.591295495	-0.73580725

Supplementary Table S3

Expression Module 1

Pathway

NAME	GS<br>follow link to MSigDB	GS DETAILS	SIZE	ES	NES	NOM p-val	FDR q-val	FWER p-val
HALLMARK_EPITHELIAL_MESENCHYMAL_TRANSITION	HALLMARK_EPITHELIAL_MESENCHYMAL_TRANSITION	Details ...	119	0.55629694	2.8440008	0	0	0
HALLMARK_TNFA_SIGNALING_VIA_NFKB	HALLMARK_TNFA_SIGNALING_VIA_NFKB	Details ...	120	0.52101225	2.6372828	0	0	0
HALLMARK_IL6_JAK_STAT3_SIGNALING	HALLMARK_IL6_JAK_STAT3_SIGNALING	Details ...	51	0.53889894	2.4437075	0	0	0
HALLMARK_INTERFERON_GAMMA_RESPONSE	HALLMARK_INTERFERON_GAMMA_RESPONSE	Details ...	102	0.4839161	2.413515	0	0	0
HALLMARK_INFLAMMATORY_RESPONSE	HALLMARK_INFLAMMATORY_RESPONSE	Details ...	116	0.47081843	2.3945246	0	0	0
HALLMARK_COAGULATION	HALLMARK_COAGULATION	Details ...	76	0.49070486	2.3592656	0	0	0
HALLMARK_APOPTOSIS	HALLMARK_APOPTOSIS	Details ...	101	0.44845754	2.2582138	0	0	0
HALLMARK_COMPLEMENT	HALLMARK_COMPLEMENT	Details ...	122	0.43771365	2.2548144	0	0	0
HALLMARK_IL2_STAT5_SIGNALING	HALLMARK_IL2_STAT5_SIGNALING	Details ...	107	0.4434922	2.234242	0	0	0
HALLMARK_HYPOXIA	HALLMARK_HYPOXIA	Details ...	111	0.44082114	2.2304697	0	0	0
HALLMARK_PROTEIN_SECRETION	HALLMARK_PROTEIN_SECRETION	Details ...	57	0.43549153	2.0029528	0	2.56E-04	0.003
HALLMARK_GLYCOLYSIS	HALLMARK_GLYCOLYSIS	Details ...	100	0.39919165	1.9832736	0	3.93E-04	0.005
HALLMARK_INTERFERON_ALPHA_RESPONSE	HALLMARK_INTERFERON_ALPHA_RESPONSE	Details ...	44	0.4466719	1.9516271	0.001011122	5.08E-04	0.007
HALLMARK_REACTIVE_OXIGEN_SPECIES_PATHWAY	HALLMARK_REACTIVE_OXIGEN_SPECIES_PATHWAY	Details ...	34	0.45772335	1.9411477	0	4.71E-04	0.007
HALLMARK_ALLOGRAFT_REJECTION	HALLMARK_ALLOGRAFT_REJECTION	Details ...	117	0.3778858	1.8965789	0	8.20E-04	0.013
HALLMARK_KRAS_SIGNALING_UP	HALLMARK_KRAS_SIGNALING_UP	Details ...	115	0.37339664	1.8736395	0	0.001007193	0.017
HALLMARK_MTORC1_SIGNALING	HALLMARK_MTORC1_SIGNALING	Details ...	112	0.3639201	1.8387457	0	0.001455937	0.026
HALLMARK_UNFOLDED_PROTEIN_RESPONSE	HALLMARK_UNFOLDED_PROTEIN_RESPONSE	Details ...	51	0.40231565	1.8295085	0.001006036	0.001481672	0.028
HALLMARK_P53_PATHWAY	HALLMARK_P53_PATHWAY	Details ...	92	0.36283466	1.7855086	0	0.002252903	0.045

Ontology

NAME	GS<br>follow link to MSigDB	GS DETAILS	SIZE	ES	NES	NOM p-val	FDR q-val	FWER p-val
REGULATION_OF_I_KAPPAB_KINASE_NF_KAPPAB_CASCADE	REGULATION_OF_I_KAPPAB_KINASE_NF_KAPPAB_CASCADE	Details ...	44	0.5697531	2.5787356	0	0	0
I_KAPPAB_KINASE_NF_KAPPAB_CASCADE	I_KAPPAB_KINASE_NF_KAPPAB_CASCADE	Details ...	57	0.5507106	2.5333748	0	0	0
POSITIVE_REGULATION_OF_I_KAPPAB_KINASE_NF_KAPPAB_CASCADE	POSITIVE_REGULATION_OF_I_KAPPAB_KINASE_NF_KAPPAB_CASCADE	Details ...	43	0.5635465	2.485007	0	0	0
POSITIVE_REGULATION_OF_SIGNAL_TRANSDUCTION	POSITIVE_REGULATION_OF_SIGNAL_TRANSDUCTION	Details ...	65	0.4820055	2.2450554	0	0.001439149	0.006
GROWTH_FACTOR_BINDING	GROWTH_FACTOR_BINDING	Details ...	15	0.6105122	2.1058583	0.001106195	0.009196991	0.046
RUFFLE	RUFFLE	Details ...	17	0.5901742	2.0956807	0	0.007983343	0.048

Comment: An indicated p-value of "0" stands for p<0.0001

Expression Module 2

Pathway

NAME	GS<br>follow link to MSigDB	GS DETAILS	SIZE	ES	NES	NOM p-val	FDR q-val	FWER p-val

LEIN_NEURON_MARKERS	LEIN_NEURON_MARKERS	Details ...	36	0.79355085	3.2301707	0	0	0
VERHAAK_GLIOBLASTOMA_NEURAL	VERHAAK_GLIOBLASTOMA_NEURAL	Details ...	76	0.6992351	3.1415334	0	0	0
REACTOME_NEURONAL_SYSTEM	REACTOME_NEURONAL_SYSTEM	Details ...	181	0.58458513	2.8434193	0	0	0
VERHAAK_GLIOBLASTOMA_PRONEURAL	VERHAAK_GLIOBLASTOMA_PRONEURAL	Details ...	90	0.6124937	2.8142054	0	0	0
REACTOME_NEUROTRANSMITTER_RELEASE_CYCLE	REACTOME_NEUROTRANSMITTER_RELEASE_CYCLE	Details ...	25	0.74820673	2.768792	0	0	0
REACTOME_TRANSMISSION_ACROSS_CHEMICAL_SYNAPSES	REACTOME_TRANSMISSION_ACROSS_CHEMICAL_SYNAPSES	Details ...	119	0.5836991	2.7493186	0	0	0
LU_AGING_BRAIN_DN	LU_AGING_BRAIN_DN	Details ...	83	0.59680474	2.7157464	0	0	0
LEE_TARGETS_OF_PTCH1_AND_SUFU_DN	LEE_TARGETS_OF_PTCH1_AND_SUFU_DN	Details ...	47	0.6485147	2.7125256	0	0	0
REACTOME_POTASSIUM_CHANNELS	REACTOME_POTASSIUM_CHANNELS	Details ...	75	0.586142	2.652736	0	0	0
KIM_ALL_DISORDERS_CALB1_CORR_UP	KIM_ALL_DISORDERS_CALB1_CORR_UP	Details ...	275	0.51495576	2.5858028	0	0	0
REACTOME_VOLTAGE_GATED_POTASSIUM_CHANNELS	REACTOME_VOLTAGE_GATED_POTASSIUM_CHANNELS	Details ...	33	0.6390277	2.5694869	0	0	0
WANG_LSD1_TARGETS_DN	WANG_LSD1_TARGETS_DN	Details ...	23	0.6803653	2.531583	0	0	0
REACTOME_ION_CHANNEL_TRANSPORT	REACTOME_ION_CHANNEL_TRANSPORT	Details ...	35	0.61844075	2.4863052	0	0	0
REACTOME_NEUROTRANSMITTER_RECEPTOR_BINDING_AND_DOWNSTREAM_TRANSMISSION_IN_THE_POSTSYNAPTIC_CELL	REACTOME_NEUROTRANSMITTER_RECEPTOR_BINDING_AND_DOWNSTREAM_TRANSMISSION_IN_THE_POSTSYNAPTIC_CELL	Details ...	86	0.53090507	2.4596374	0	0	0
MCCLUNG_DELTA_FOSB_TARGETS_8WK	MCCLUNG_DELTA_FOSB_TARGETS_8WK	Details ...	25	0.64918005	2.451265	0	0	0
MCCLUNG_CREB1_TARGETS_UP	MCCLUNG_CREB1_TARGETS_UP	Details ...	45	0.5760586	2.4475546	0	0	0
LEIN_OLIGODENDROCYTE_MARKERS	LEIN_OLIGODENDROCYTE_MARKERS	Details ...	32	0.60218614	2.432109	0	0	0
REACTOME_POST_NMDA_RECEPTOR_ACTIVATION_EVENTS	REACTOME_POST_NMDA_RECEPTOR_ACTIVATION_EVENTS	Details ...	22	0.6431489	2.3713114	0	0	0
ASTON_MAJOR_DEPRESSIVE_DISORDER_DN	ASTON_MAJOR_DEPRESSIVE_DISORDER_DN	Details ...	86	0.5089443	2.3656058	0	0	0

Ontology

NAME	GS<br>follow link to MSigDB	GS DETAILS	SIZE	ES	NES	NOM p-val	FDR q-val	FWER p-val
VOLTAGE_GATED_CATION_CHANNEL_ACTIVITY	VOLTAGE_GATED_CATION_CHANNEL_ACTIVITY	Details ...	43	0.59378016	2.4700959	0	0	0
SYNAPTIC_TRANSMISSION	SYNAPTIC_TRANSMISSION	Details ...	105	0.52101654	2.4363241	0	0	0
POTASSIUM_ION_TRANSPORT	POTASSIUM_ION_TRANSPORT	Details ...	44	0.5752327	2.4193408	0	0	0
VOLTAGE_GATED_CHANNEL_ACTIVITY	VOLTAGE_GATED_CHANNEL_ACTIVITY	Details ...	48	0.5620596	2.3980422	0	2.23E-04	0.001
VOLTAGE_GATED_POTASSIUM_CHANNEL_COMPLEX	VOLTAGE_GATED_POTASSIUM_CHANNEL_COMPLEX	Details ...	27	0.6246317	2.3750608	0	1.78E-04	0.001
TRANSMISSION_OF_NERVE_IMPULSE	TRANSMISSION_OF_NERVE_IMPULSE	Details ...	114	0.49893552	2.3531435	0	1.48E-04	0.001
VOLTAGE_GATED_POTASSIUM_CHANNEL_ACTIVITY	VOLTAGE_GATED_POTASSIUM_CHANNEL_ACTIVITY	Details ...	25	0.62578964	2.3481867	0	1.27E-04	0.001
ION_CHANNEL_ACTIVITY	ION_CHANNEL_ACTIVITY	Details ...	87	0.5044314	2.3469286	0	1.11E-04	0.001
GATED_CHANNEL_ACTIVITY	GATED_CHANNEL_ACTIVITY	Details ...	72	0.521216	2.3463588	0	9.89E-05	0.001
POTASSIUM_CHANNEL_ACTIVITY	POTASSIUM_CHANNEL_ACTIVITY	Details ...	36	0.5776478	2.3348494	0	8.91E-05	0.001
METAL_ION_TRANSPORT	METAL_ION_TRANSPORT	Details ...	75	0.5076481	2.3138921	0	8.10E-05	0.001
MONOVALENT_INORGANIC_CATION_TRANSPORT	MONOVALENT_INORGANIC_CATION_TRANSPORT	Details ...	62	0.5307589	2.3061347	0	7.42E-05	0.001
CATION_CHANNEL_ACTIVITY	CATION_CHANNEL_ACTIVITY	Details ...	71	0.5095651	2.2474594	0	6.85E-05	0.001
METAL_ION_TRANSMEMBRANE_TRANSPORTER_ACTIVITY	METAL_ION_TRANSMEMBRANE_TRANSPORTER_ACTIVITY	Details ...	87	0.486302	2.2306135	0	6.36E-05	0.001
SUBSTRATE_SPECIFIC_CHANNEL_ACTIVITY	SUBSTRATE_SPECIFIC_CHANNEL_ACTIVITY	Details ...	91	0.47928095	2.205262	0	1.19E-04	0.002
CATION_TRANSPORT	CATION_TRANSPORT	Details ...	96	0.46490255	2.1780188	0	2.23E-04	0.004
REGULATION_OF_NEUROTRANSMITTER_LEVELS	REGULATION_OF_NEUROTRANSMITTER_LEVELS	Details ...	15	0.64841914	2.1598208	0.001102536	5.25E-04	0.01
NEUROTRANSMITTER_RECEPTOR_ACTIVITY	NEUROTRANSMITTER_RECEPTOR_ACTIVITY	Details ...	29	0.5460957	2.1180286	0	8.41E-04	0.017
ION_TRANSPORT	ION_TRANSPORT	Details ...	117	0.44581825	2.1152372	0	9.37E-04	0.02
NEUROTRANSMITTER_BINDING	NEUROTRANSMITTER_BINDING	Details ...	31	0.5380597	2.1091177	0	0.001023261	0.023
CATION_TRANSMEMBRANE_TRANSPORTER_ACTIVITY	CATION_TRANSMEMBRANE_TRANSPORTER_ACTIVITY		127	0.4344515	2.0866168	0	0.001610888	0.036

Expression Module 3

Pathway

NAME	GS<br>follow link to MSigDB	GS DETAILS	SIZE	ES	NES	NOM p-val	FDR q-val	FWER p-val
VERHAAK_GLIOBLASTOMA_PRONEURAL	VERHAAK_GLIOBLASTOMA_PRONEURAL	Details ...	90	0.7009721	3.6541247	0	0	0
VERHAAK_GLIOBLASTOMA_NEURAL	VERHAAK_GLIOBLASTOMA_NEURAL	Details ...	76	0.5518531	2.7807393	0	0	0
LEIN_NEURON_MARKERS	LEIN_NEURON_MARKERS	Details ...	36	0.5819332	2.5886965	0	0	0
REACTOME_NEURONAL_SYSTEM	REACTOME_NEURONAL_SYSTEM	Details ...	181	0.45277166	2.5045533	0	0	0
LU_AGING_BRAIN_DN	LU_AGING_BRAIN_DN	Details ...	83	0.48975444	2.4855797	0	0	0

Ontology

NAME	GS<br>follow link to MSigDB	GS DETAILS	SIZE	ES	NES	NOM p-val	FDR q-val	FWER p-val
SYNAPTIC_TRANSMISSION	SYNAPTIC_TRANSMISSION	Details ...	105	0.43119714	2.2886755	0	0.006722231	0.007
TRANSMISSION_OF_NERVE_IMPULSE	TRANSMISSION_OF_NERVE_IMPULSE	Details ...	114	0.4159442	2.2452185	0	0.007690805	0.016
VOLTAGE_GATED_CATION_CHANNEL_ACTIVITY	VOLTAGE_GATED_CATION_CHANNEL_ACTIVITY	Details ...	43	0.48607305	2.2200336	0	0.007685192	0.024
VOLTAGE_GATED_CHANNEL_ACTIVITY	VOLTAGE_GATED_CHANNEL_ACTIVITY	Details ...	48	0.47953898	2.211258	0	0.005763894	0.024
